# Role of Aspartic and Polyaspartic Acid on the Synthesis and Hydrolysis of Brushite

**DOI:** 10.3390/jfb10010011

**Published:** 2019-02-01

**Authors:** Katia Rubini, Elisa Boanini, Adriana Bigi

**Affiliations:** Department of Chemistry “Giacomo Ciamician”, Alma Mater Studiorum-University of Bologna, 40126 Bologna, Italy; katia.rubini@unibo.it

**Keywords:** dicalcium phosphate dihydrate, calcium phosphate, crystals, aspartic acid, poly-aspartic acid, hydrolysis, heat treatment, phase conversion, X-ray diffraction, bone cements

## Abstract

Dicalcium phosphate dihydrate (DCPD) is one of the mineral phases indicated as possible precursors of biological apatites and it is widely employed in the preparation of calcium phosphate bone cements. Herein, we investigated the possibility to functionalize DCPD with aspartic acid (ASP) and poly-aspartic acid (PASP), as models of the acidic macromolecules of biomineralized tissues, and studied their influence on DCPD hydrolysis. To this aim, the synthesis of DCPD was performed in aqueous solution in the presence of increasing concentrations of PASP and ASP, whereas the hydrolysis reaction was carried out in physiological solution up to three days. The results indicate that it is possible to prepare DCPD functionalized with PASP up to a polyelectrolyte content of about 2.3 wt%. The increase of PASP content induces crystal aggregation, reduction of the yield of the reaction and of the thermal stability of the synthesized DCPD. Moreover, DCPD samples functionalized with PASP display a slower hydrolysis than pure DCPD. On the other hand, in the explored range of concentrations (up to 10 mM) ASP is not incorporated into DCPD and does not influence its crystallization nor its hydrolysis. At variance, when present in the hydrolysis solution, ASP, and even more PASP, delays the conversion into the more stable phases, octacalcium phosphate and/or hydroxyapatite. The greater influence of PASP on the synthesis and hydrolysis of DCPD can be ascribed to the cooperative action of the carboxylate groups and to its good fit with DCPD structure.

## 1. Introduction

The biomineralization processes of the hard tissues of vertebrates imply the deposition of calcium phosphate in an environment rich in acidic macromolecules. The inorganic phase is identified as a basic calcium phosphate, which is usually described as close to the synthetic hydroxyapatite (HA). However, it is generally accepted that precipitation of biological apatites occurs through intermediate steps, involving transformation from less stable phosphates, including amorphous calcium phosphate (ACP), dicalcium phosphate dihydrate (DCDP), tricalcium phosphate (TCP) and octacalcium phosphate (OCP) [1,2,3]. In particular, DCPD is often found in pathological calcifications and has been recently identified as an HA precursor during the mineralization of osteoblast in vitro [4]. 

The DCPD chemical formula is CaHPO_4_·2H_2_O that corresponds to the mineral Brushite, which displays the same monoclinic structure, Ia space group. The structure involves parallel CaP chains with interlayered lattice water molecules. Loss of structural water by heat treatment at about 180 °C provokes DCPD transformation into its anhydrous form, CaHPO_4_ (dicalcium phosphate anhydrous, DCPA) [5]. DCPD is stable at a relatively low pH, and in water it undergoes a transformation into the more stable phases, OCP and HA [6]. The presence of additives and the applied conditions can influence and mediate the phase transformation [7,8,9].

The biological environment of the deposition and possible transformation of the inorganic phase of the hard tissue of vertebrates is rich in proteins containing significant amounts of acidic amino acids, such as aspartic (ASP) and glutamic (GLU) acids. This has stimulated a lot of research on the nucleation and growth of HA in the presence of acidic amino acids, as well as of poly-amino acids and peptides rich in groups that are abundant in the proteins of the mineralized tissues. The results of these studies generally indicate that the presence of acidic amino acids in solution is able to inhibit HA nucleation and growth, with a concentration modulated effect, although to a lower extent than poly-amino acids and proteins [10,11,12,13,14]. Investigations on the role of these additives on the nucleation and growth of the calcium phosphates indicated as possible precursors of biological apatites report similar results. In particular, it was found that PASP inhibition of DCPD crystal growth occurs through interaction of the poly-amino acid with the (010) faces of the structure [15], whereas its interaction with OCP takes place through the hydrated layer of the unit cell [16]. On the other hand, low concentrations of glutamic and aspartic acid showed no significant effect on the rate of growth and growth morphology of the DCPD crystals [15]. At variance, the presence of relatively high concentrations (30 mM) of aspartic acid during precipitation of calcium phosphates through the vapor diffusion sitting drop micro-method has been reported to lead to crystallization of DCPD [17].

In order to clarify the role of acidic amino and poly-amino acids on the synthesis and hydrolysis of DCPD, we investigated the structural and morphological modifications induced by the presence of aspartic acid (ASP) and poly-aspartic acid (PASP) during the synthesis, as well as the hydrolysis, of DCPD.

## 2. Materials and Methods

### 2.1. Synthesis and Hydrolysis Procedures

Synthesis of brushite (DCPD) was carried out using 150 mL of a phosphate solution containing 5 mmol of Na_2_HPO_4_·12H_2_O (Carlo Erba Reagents, Milano, Italy) and 5 mmol of NaH_2_PO_4_·H_2_O (Carlo Erba Reagents, Milano, Italy), pH 4.90 adjusted with glacial CH_3_COOH. The solution was heated at 37 °C and 50 mL of 0.2 M Ca(CH_3_COO)_2_·H_2_O (Carlo Erba Reagents, Milano, Italy) was added drop-wise over a period of 15 min, under continuous stirring. Afterwards the precipitate was stored in contact with the mother solution for 10 min, filtered, repeatedly washed with bi-distilled water and dried at 37 °C.

Syntheses in the presence of the Sodium Polyaspartate (MW 11,000, Sigma Aldrich, Milano, Italy) or Aspartic Acid (Sigma Aldrich, Milano, Italy) were carried out by following the same procedure and inserting the additive in the phosphate solution before pH correction. PASP concentrations calculated on the final volume were 0.2, 0.5, 0.8, and 1 mM (in monomer), thereby samples were labelled PASP02, PASP05, PASP08, and PASP1, respectively. ASP concentrations calculated on final volume were 1, 2, 5, and 10 mM, and samples were labelled ASP1, ASP2, ASP5, and ASP10, respectively.

Heat treatment of all samples synthesized in the absence and in the presence of various concentrations of PASP or ASP was performed at 300 °C for 30 min.

Hydrolysis of DCPD was carried out in physiological solution (NaCl 0.9%) or in physiological solution containing PASP 0.8 mM in monomer (inPASP08) or ASP 10 mM (inASP10). In addition, brushitic samples synthesized in the presence of PASP and ASP (PASP08 and ASP10) were submitted to incubation in a physiological solution. The reactions of hydrolysis were performed on 100 mg of powder sample incubated into 25 mL solution at 37 °C or 60 °C, up to 72 h. The final solid products were centrifuged at 10,000 rpm for 15 min, then dried at 37 °C.

### 2.2. Characterization

X-ray diffraction analysis was carried out by means of a PANalytical X’Pert PRO powder diffractometer (Malvern Panalytical, Milano, Italy) equipped with a fast X’Celerator detector (Malvern Panalytical, Milano, Italy). CuKα radiation was used (40 mA, 40 kV). The 2θ range was investigated from 3 to 60 2θ degrees with a step size of 0.1° and time/step of 100 s. For FT-IR adsorption analysis, about 1 mg of the powdered samples was carefully mixed with KBr (200 mg, infrared grade) and pelletized under a pressure of 10 tons for 2 min. The pellets were analyzed using a Bruker ALPHA FT-IR spectrophotometer (Bruker Italia, Milano, Italy) to collect 32 scans in the range 4000–400 cm^−1^ at a resolution of 4 cm^−1^.

Calorimetric measurements were performed using a Perkin Elmer Pyris Diamond differential scanning calorimeter (Perkin Elmer, Waltham, MA, USA) equipped with a model ULSP 90 intra-cooler (Perkin Elmer, Waltham, MA, USA). Heating was carried out in aluminum open pan at 5 °C min^−1^ in the temperature range from 50 °C to 215 °C.

Elemental analysis of light elements was performed on a Thermo Flash 2000 CHNS/O Analyzer (Thermo Fisher, Waltham, MA, USA). Each sample was weighed in tin capsules, then dropped into an oxidation/reduction reactor kept at a temperature of 900–1000 °C. Produced gases were separated in a chromatographic column and detected by a highly sensitive thermal conductivity detector (Thermo Fisher, Waltham, MA, USA).

Zeta potential was measured using a Malvern Instruments Zetasizer Nano (Malvern Panalytical, Milano, Italy). 5 mg of powder sample was suspended in 50 mL of MilliQ water after sonication for 2 min.

Morphological investigation was performed using a HITACHI S-2400 scanning electron microscope (Hitachi Ltd., Tokio, Japan) operating at 15 kV. The samples were sputter-coated with gold before examination.

## 3. Results

### 3.1. Synthesis of DCPD in the Presence of PASP

**Figure 1:**
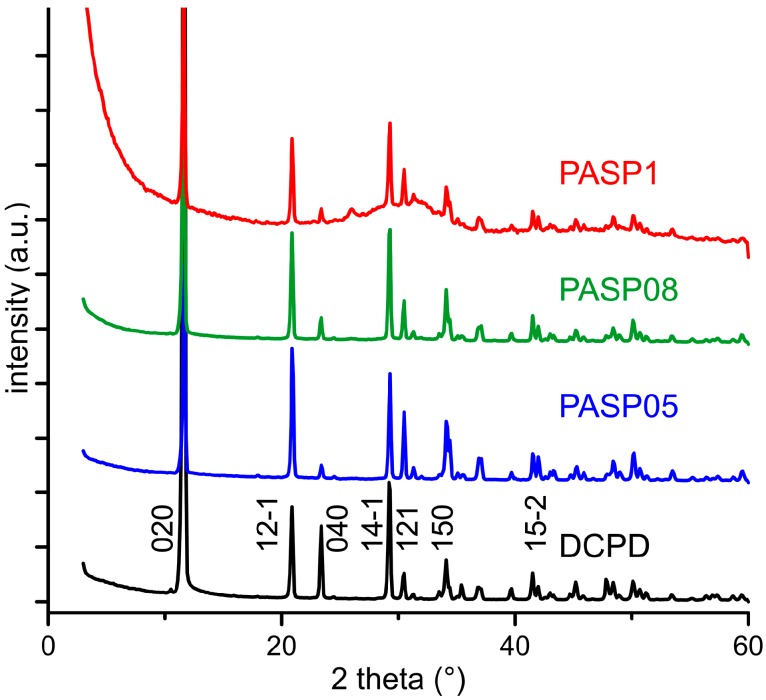
Powder X-ray diffraction patterns of the products obtained from the synthesis of dicalcium phosphate dihydrate (DCPD) in the presence of increasing concentrations of PASP. The indexes of the main reflections of DCPD are indicated (PDF 01-072-0713).

The powder X-ray diffraction patterns of the products obtained from the synthesis of DCPD in the presence of increasing concentrations of PASP are reported in Figure 1. The patterns show a series of diffraction reflections consistent with the presence of DCPD as unique crystalline phase up to a poly-amino acid concentration in solution of 0.8 mM. However, the significant decrease of the amount of precipitate on increasing PASP concentration (Table 1) indicates an inhibitory role of the poly-amino acid on the crystallization of DCPD.

Increasing the concentration up to 1 mM provokes the appearance of a broad halo superimposed to the reflections of DCPD, suggesting the presence of amorphous material.

**Figure 2:**
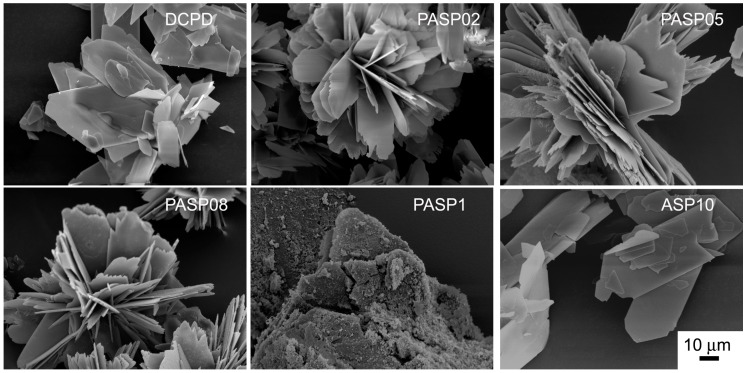
Scanning Electron Microscope images of the products obtained from the synthesis of DCPD in the presence of PASP or ASP. Magnification in all the images is the same for direct comparison.

Further increase of PASP up to 1.5 and 2 mM provides products that show XRD patterns devoid of diffraction reflections and consistent with the presence of amorphous material (data not shown). The patterns of PASP02, PASP05, and PASP08 display a slightly modified distribution of the relative intensity of the diffraction peaks when compared to pure DCPD. The most evident variation is the increase of the relative intensity of the 040 reflection (2θ = 23.4°), which can be justified by the orientation effect and morphology variation [18]. PASP has indeed a great effect on the morphology of the precipitates. Scanning Electron Microscopy (SEM) images reported in Figure 2 show that pure DCPD is constituted of plate like crystals with large (0k0) faces and sharp edges, whereas the crystals synthesized in the presence of PASP up to PASP08 exhibit indented edges and a strong tendency to aggregate into spherulites. The crystallization of the material precipitated in the presence of 1 mM PASP is so disturbed that the presence of crystals is barely appreciable in its SEM image (Figure 2).

The plate-like DCPD crystals tend to lie flat on the sample holder of the diffractometer, with consequent enhancement of the relative intensity of the 0k0 reflections. Crystal aggregation of the products synthesized in the presence of PASP reduces this phenomenon and, as a consequence, the relative intensity of the relevant X-ray diffraction (XRD) reflections.

**Figure 3:**
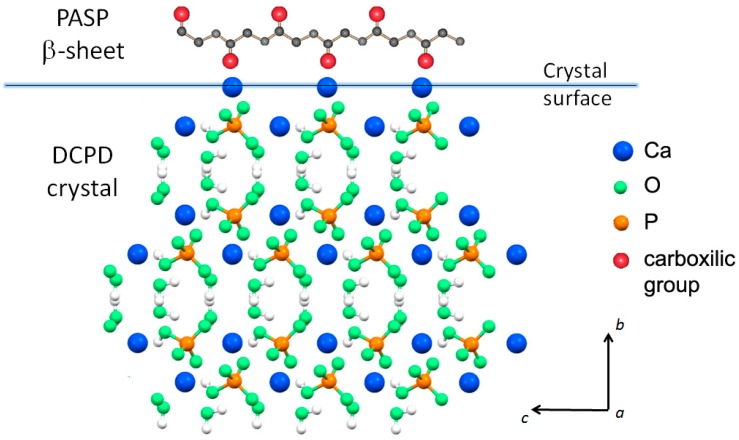
Possible interaction between carboxylic groups in the polyaspartic β-sheet and the calcium ions exposed on the large DCPD crystal face ((0k0) plane).

Possible interaction between PASP and the (0k0) faces of DCPD crystals would be favored by their good structural fit: The distances between neighboring calcium ions on the (0k0) faces along the crystallographic *a* direction (6.23 Å) are commensurate to those between carboxylic groups in the polyaspartic β-sheet (6.90 Å). On considering that PASP in solution has not a rigid structure and the modest difference of distances, it is possible to suggest interactions between the carboxylic groups perpendicular to the crystal flat plane ((0k0) plane) and the calcium ions exposed on the large crystal face. This interaction model (Figure 3) is much more convincing than that previously advanced on the basis of the distances between neighboring calcium ions from two adjacent layers within one Ca-HPO4 bilayer (parallel to the (0k0) plane), which were erroneously reported as 6.95 Å [15].

**Figure 4:**
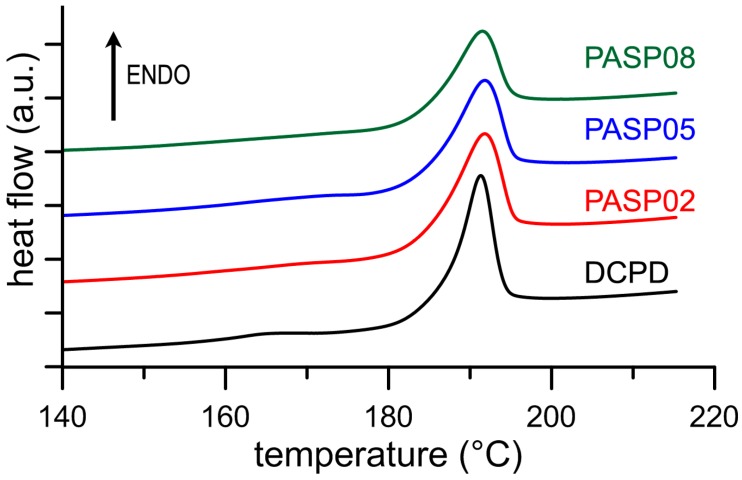
DSC plots showing the thermal behavior of the products obtained from the synthesis of DCPD in the presence of increasing concentrations of PASP.

The results of the Differential Scanning Calorimetry (DSC) analysis put into evidence the influence of the polyelectrolyte on the stability of DCPD. In fact, although the endothermic peak corresponding to the transformation of the DCPD into DCPA is displayed at about 190 °C for all the samples (Figure 4), the values of ΔH associated to this process decreases significantly as a function of PASP concentration in solution, as shown in Table 1. The data indicate that the energy required to transform DCPD into its anhydrous form decreases on increasing the presence of PASP in the reaction medium, which suggests a destabilization of DCPD structure provoked by the poly-amino acid.

The FT-IR spectrum of DCPD shows the presence of a number of absorption bands due to H_2_O stretching (3000–3500 cm^−1^) and bending (1651 cm^−1^), PO stretching (900–1300 cm^−1^) and bending (500–700 cm^−1^) modes, as well as to P-O(H) stretching at about 876 cm^−1^ (Figure S1) [8,19]. Sample PASP08 displays a FT-IR spectrum which shows, other than the characteristic bands of DCPD, a shoulder at about 1580 cm^−1^ and an absorption band at about 1410 cm^−1^ indicating the presence of the polyelectrolyte, as it can be inferred by comparing the spectrum with that of PASP (Figure S1). The presence of the polyelectrolyte in the samples is further supported by the values of zeta potential, which are more negative for the products synthesized in the presence of PASP than that measured for pure DCPD (Table 1).

Moreover, the results of elemental analysis indicate a significant content of carbon, which increases with PASP concentration in solution and allows us to calculate that the samples contain up to about 2.3 wt% of the poly-amino acid, as reported in Table 1.

In agreement, after heat treatment at 300 °C, the PASP containing powders display a pale-yellow color, which becomes darker on passing from PASP02 to PASP08 (Figure S2), due to the partial combustion of organic material and residual C remains.

**Figure 5:**
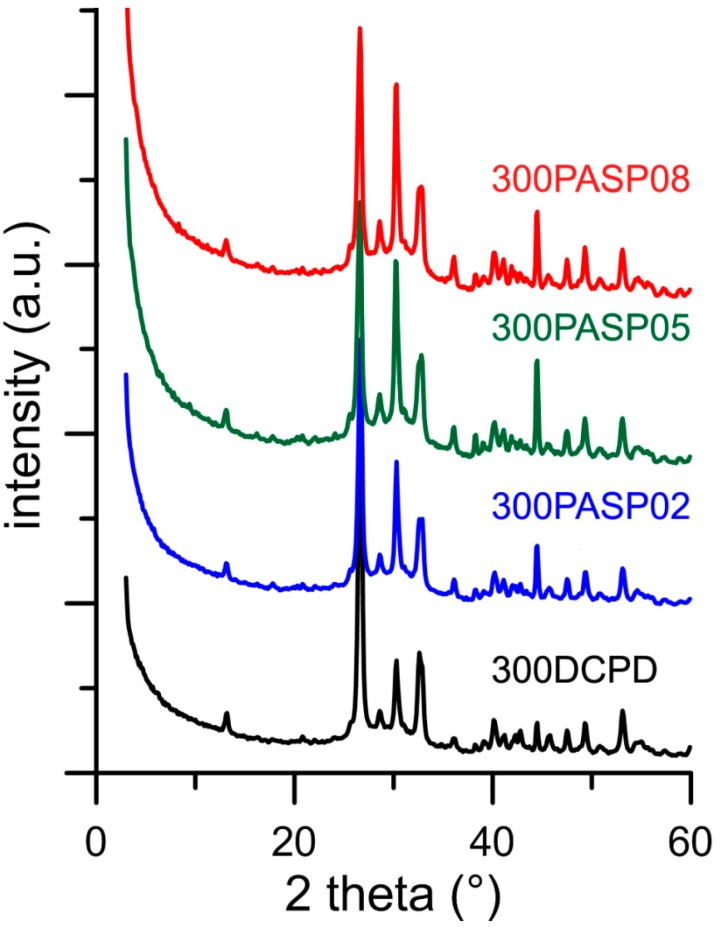
Powder X-ray diffraction patterns of the products obtained from the synthesis of DCPD in the presence of increasing concentrations of PASP after heat treatment at 300 °C.

After heat treatment, all the samples exhibit XRD patterns consistent with the presence of DCPA as unique crystalline phase (Figure 5). In addition, after heat treatment the patterns of the PASP functionalized samples present a slightly different distribution of the relative intensity of the reflections in comparison with that of the sample obtained by heat treatment of pure DCPD.

**Figure 6:**
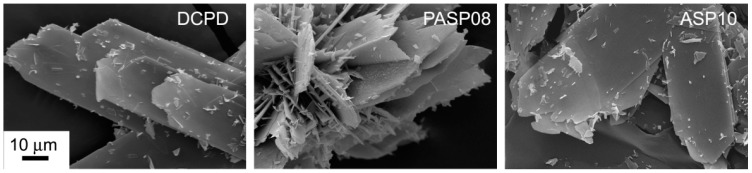
SEM images of DCPD, PASP08 and ASP10 crystals after heat treatment at 300 °C. Magnification in all the images is the same for direct comparison.

The differences can be ascribed to the different morphology of the samples: 300DCPD maintains the characteristic plate-like morphology, which can result in crystal orientation, whereas 300PASP samples are still aggregated into spherulites (Figure 6). Moreover, all the SEM images show the presence of a quantity of small particles on the surface of the crystals, most likely due to a partial crumbling of the crystal during dehydration caused by heat treatment.

### 3.2. Synthesis of DCPD in the Presence of ASP

**Figure 7:**
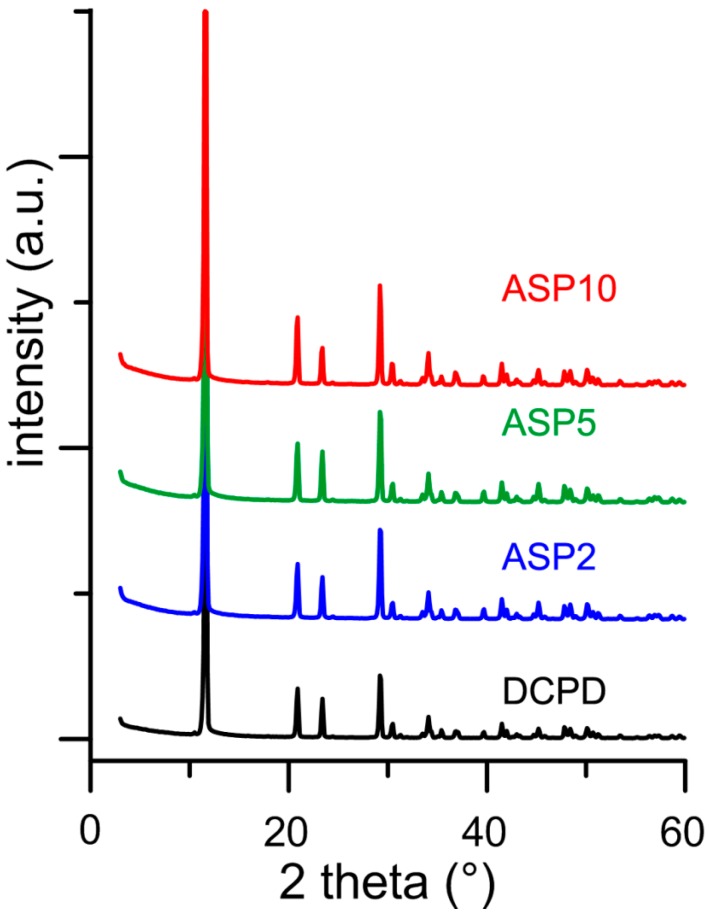
Powder X-ray diffraction patterns of the products obtained from the synthesis of DCPD in the presence of increasing concentrations of ASP.

In contrast with what was found for the influence of PASP on the synthesis of DCPD, all the results obtained on the products of synthesis in the presence of ASP indicate that the amino-acid does not cause any structural or morphological modification and its presence is not detectable in the precipitates. In fact, all these products exhibit XRD patterns consistent with the presence of DCPD as a unique crystalline phase (Figure 7). The distribution of the relative intensity of the diffraction peaks appears similar to that of pure DCPD. In agreement, SEM images show the characteristic morphology of the DCPD crystals (Figure 2).

Moreover, the values of ΔH associated to the phase transformation from DCPD to DCPA do not vary with ASP concentration and are very close to those of pure DCPD (Table 1, Figure S3).

The data of zeta potential further support the absence of the amino acid in the products of synthesis (Table 1), which is confirmed also by the white color of the samples heat treated at 300 °C. The XRD patterns of heat-treated samples indicate that they are constituted by DCPA (Figure S4).

In agreement with the results obtained on prepared samples, the morphology of the crystals and the relative intensity distributions of the XRD peaks recorded from 300ASP samples do not differ significantly from that of 300DCPD (Figure 6 and Figure S4).

It was previously reported that aspartic acid can be incorporated into HA crystals if present in the synthesis solution [20]. The different effect of ASP on the synthesis of HA and DCPD might be ascribed to a better structural interaction of the amino acid with HA as suggested by the variation of the mean dimensions of the perfect crystalline domains of HA, which indicates a preferential interaction of ASP with specific crystallographic faces of HA [20].

### 3.3. Hydrolysis

The analysis of the samples stored in physiological solution for different periods of time up to 3 days puts into evidence the different roles played by the additives and by temperature. On comparing the results obtained when the hydrolysis is carried out at 37 °C with those at 60 °C, it is evident that increasing the temperature accelerates the transformation of DCPD into thermodynamically more stable phases, in agreement with previous data [21,22].

At 37 °C pure DCPD is partially transformed into OCP after just 3 h (the conversion into OCP is complete after 2 days) and at 3 days HA appears as a secondary phase; at 60 °C HA is present since the first stages, and it is the only crystalline phase after 2 days (Table 2 and Table 3).

The data obtained for PASP08 confirm the presence of the poly-amino acid in the sample. In fact, the phase conversion is delayed when compared to that observed for pure DCPD: After 3 days at 37 °C just a part of DCPD is converted into OCP, whereas at 60 °C the phases obtained after 3 days are OCP and HA. On the contrary, the hydrolysis of ASP10 proceeds with the same trend as that of pure DCPD, in agreement with the absence of aspartic acid in the sample.

**Figure 8:**
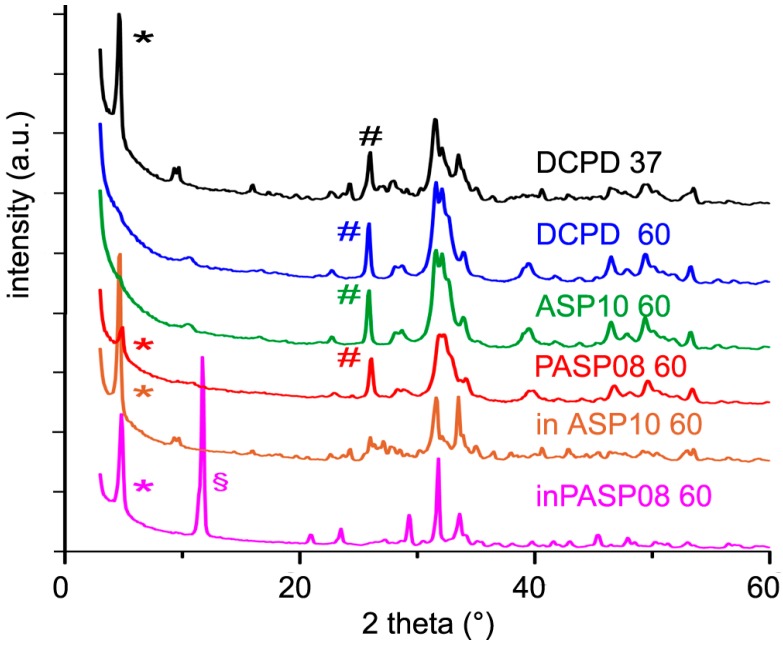
Powder X-ray diffraction patterns of the products obtained from the hydrolysis of DCPD, PASP08, and ASP10 in physiological solution and of DCPD in the presence of PASP (inPASP08) or ASP (inASP10) after 3 days at 37 or 60 °C. The symbols (§), (*) and (#) indicate reflections diagnostic of DCPD, OCP, and HA, respectively.

Furthermore, the presence in the hydrolysis solution of PASP or ASP results in a strong inhibition of the conversion of DCPD, as shown by the data reported in Table 2 and Table 3 and by comparing the XRD patterns recorded from the different samples after three days at 60 °C (Figure 8). PASP is even more effective than ASP in delaying the hydrolysis reaction, thus that the pattern still shows the presence of DCPD even after 3 days at 60 °C.

The presence of the different phases can be appreciated also by comparing the FT-IR spectra of the samples after 3 days of hydrolysis at 60 °C (Figure S5). In particular, the absence of the bands at 3572 and 630 cm^−1^, due to OH stretching and bending modes respectively, in the spectra of DCPD and ASP10 confirms the poor crystallinity of the apatitic phases obtained from their hydrolysis. The spectrum of inASP after hydrolysis shows the characteristic bands of OCP [23], whereas that of inPASP exhibits a greater number of bands due to the co-presence of OCP and DCPD (Figure S5). Moreover, the band at about 1410 cm^−1^ in the spectra of PASP08 and inPASP after hydrolysis indicates the presence of the polyelectrolyte in these samples.

**Figure 9:**
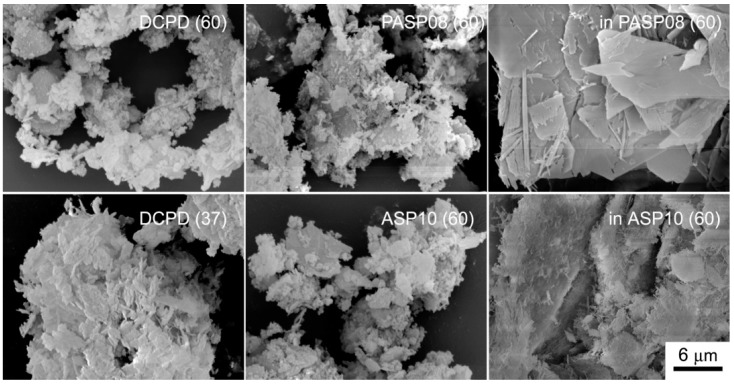
SEM images of the products obtained from the hydrolysis reaction of DCPD, PASP08 and ASP10 in physiological solution and of DCPD in the presence of PASP (inPASP08) or ASP (inASP10) after 3 days at 37 or 60 °C. Magnification in all the images is the same for direct comparison.

The differences among the products of hydrolysis are well evident in comparing the SEM images reported in Figure 9: The big crystals of DCPD are still evident in the image of inPASP08, whereas the samples constituted of OCP and/or HA show the presence of platelet-like and/or needle crystals.

The stronger inhibition exerted by the poly-amino acid is not surprising and can be ascribed to the cooperative action of the carboxylate groups and to its good structural fit with DCPD, which promotes PASP adsorption on the surface of the (0k0) faces [15]. A similar mechanism was previously reported for the inhibition effect of PASP on OCP hydrolysis, where adsorption of the polyelectrolyte on the hydrated layer of the OCP (100) faces prevents OCP transformation into HA [24].

## 4. Conclusions

The results of this work highlight the different influences of PASP and ASP on the synthesis and hydrolysis of DCPD. PASP inhibits the crystallization of DCPD, which is completely hindered when the concentration of the polyelectrolyte exceeds 1 mM in monomeric units. At lower concentrations, PASP reduces the yield of the reaction and provokes aggregation of the DCPD crystals, most likely through interaction with the (0k0) faces. Functionalization with PASP up to about 2.3 wt% reduces the stability of the DCPD structure, thus that the energy required for its thermal transition into DCPA decreases as PASP content increases. On the contrary, the presence in solution of ASP, up to 10 mM does not affect the structure, as well the morphology and stability, of the products of synthesis of the DCPD.

The results obtained on the synthesis of the DCPD are supported by those recorded on the hydrolysis reaction. In fact, in physiological solution, the products synthesized in the presence of ASP exhibit the same extent of conversion into the more stable phases, OCP and/or HA, as that observed for pure DCPD. At variance, the samples containing PASP are characterized by a delayed conversion in solution.

ASP and PASP also maintain a different behavior when dissolved in the hydrolysis solution: both delay the phase transition, but PASP exerts a stronger inhibition than ASP.

DCPD is widely applied in the composition of calcium phosphate cements (CPCs), which are based on the hardening of the cement paste during the conversion of calcium phosphates into thermodynamically more stable phases [25,26]. It follows that the data of the present work not only provides useful information for a better understanding of the biomineralization processes but can also be exploited to modulate the hardening reaction of CPCs through the involvement of ASP and PASP in their composition.

## Figures and Tables

**Table 1:** Values of masses, ΔH of the endothermic transition from DCPD to DCPA, zeta potential and poly-aspartic acid (PASP) content of the products of syntheses.

Sample	Mass (g)	ΔH (J/g)	Zeta Potential (mV)	PASP Content (wt%)
DCPD	1.146	400	−12.0	---
PASP0.2	0.691	326	−23.0	0.4
PASP0.5	0.481	302	−21.1	1.0
PASP0.8	0.220	216	−23.3	2.3
ASP5	1.151	406	−11.6	---
ASP10	1.134	400	−12.5	---

**Table 2:** Crystalline phases present in the powder X-ray diffraction patterns of the products obtained after different periods of brushitic samples storage solution at 37 °C. D = DCPD; O = OCP; H = HA.

T = 37 °C	3 h	6 h	9 h	15 h	24 h	48 h	72 h
DCPD	D + O	D + O	D + O	D + O	D + O	O	O + H
PASP08	D + O	D + O	D + O	D + O	D + O	D + O	D + O
inPASP08	D	D	D	D	D	D	D + O
ASP10	D + O	D + O	D + O	D + O	D + O	O	O + H
inASP10	D	D	D	D	D	D	D + O

**Table 3:** Crystalline phases present in the powder X-ray diffraction patterns of the products obtained after different periods of brushitic samples storage solution at 60 °C. D = DCPD; O = OCP; H = HA.

T = 60 °C	3 h	6 h	9 h	15 h	24 h	48 h	72 h
DCPD	O + D + H	O + H	O + H	O + H	H + O	H	H
PASP08	D + O + H	D + O + H	D + O + H	H + O + D	H + O + D	H + O + D	H + O
inPASP08	D + OCP	D + O	D + O	D + O	O + D	O + D	O + D
ASP10	O + D + H	O + H	O + H	O + H	H + O	H	H
inASP10	D + O	O + D	O	O + D	O	O	O

## Supplementary Materials

The following are available online at https://www.mdpi.com/2079-4983/10/1/11/s1, Figure S1: FT-IR spectra of selected samples; Figure S2: Photograph of heat-treated samples, Figure S3: DSC plots of ASP powders; Figure S4: XRD patterns of heat-treated samples; Figure S5: FT-IR spectra of the samples after three days of hydrolysis at 60 °C.
