# Supplementary material for: Role of Aspartic and Polyaspartic Acid on the Synthesis and Hydrolysis of Brushite
Source: J Funct Biomater. 2019 Feb 1;10(1):11. doi: 10.3390/jfb10010011 (PMC6463188; doi:10.3390/jfb10010011)
Supplement: Supplementary file 1 [file jfb-10-00011-s001.pdf]

Supplementary Information

Role of aspartic and polyaspartic acid on the synthesis and hydrolysis of brushite.

Katia Rubini, Elisa Boanini and Adriana Bigi

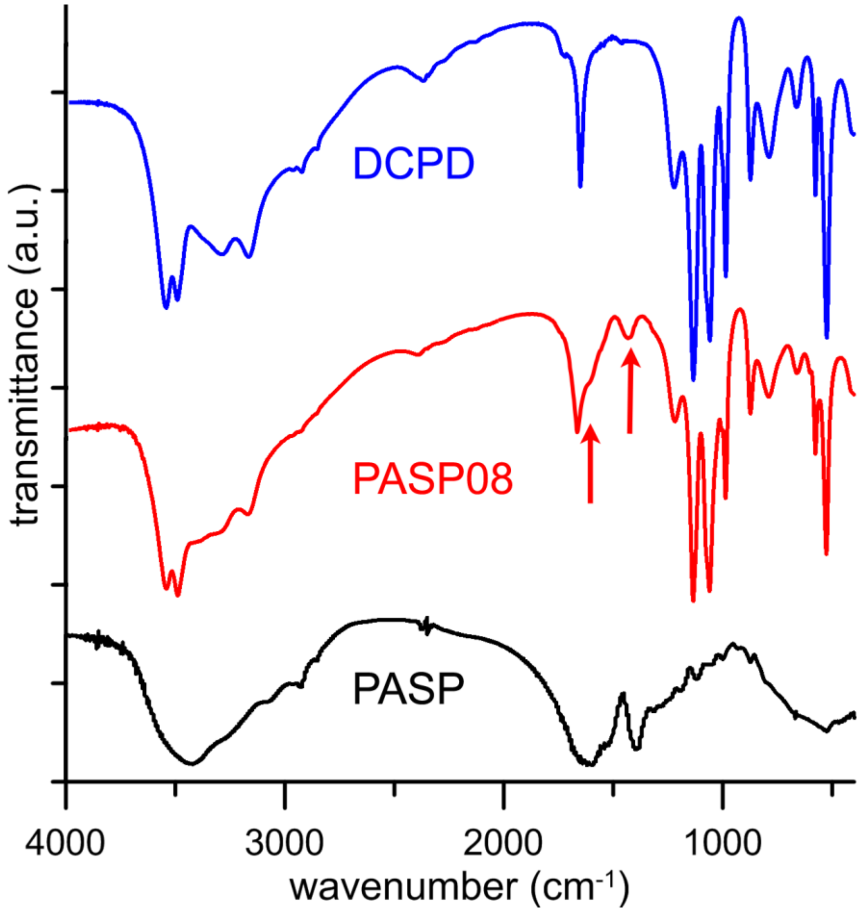

Figure S1: FT-IR spectra of the DCPD and PASP08 synthesized samples, compared with that of PASP powder.

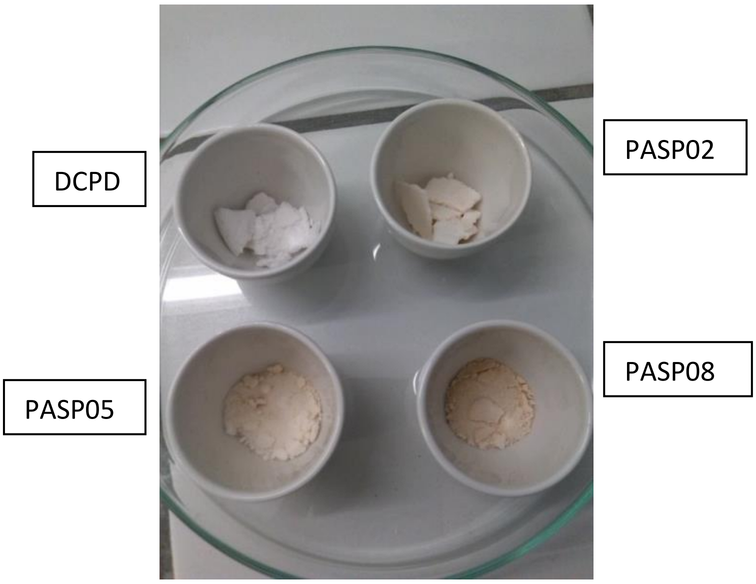

Figure S2: picture of the samples obtained from the synthesis of DCPD in the presence of increasing concentrations of PASP after heat treatment at 300 °C. Powders display a pale yellow color, which becomes darker on passing from PASP02 to PASP08, due to the partial combustion of organic material and residual C remains.

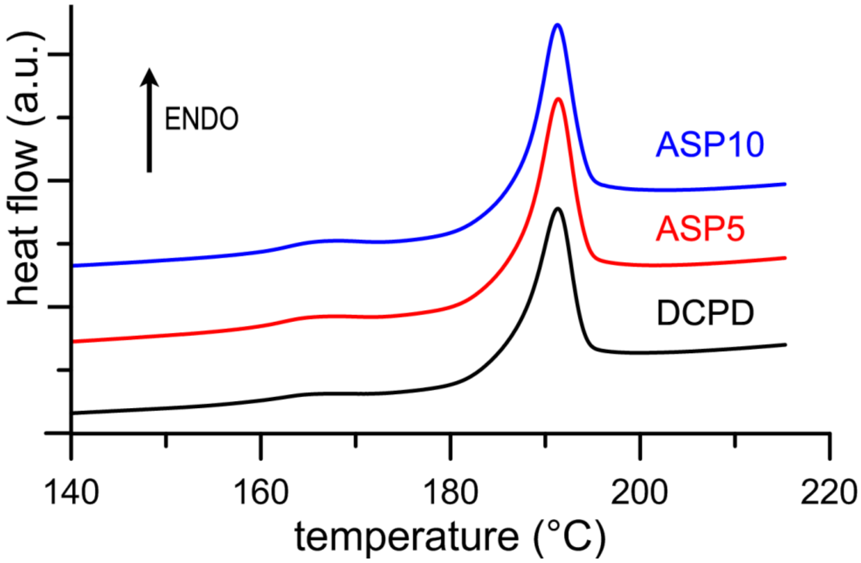

Figure S3: DSC plots showing the thermal behavior of the products obtained from the synthesis of DCPD in the presence of increasing concentrations of ASP.

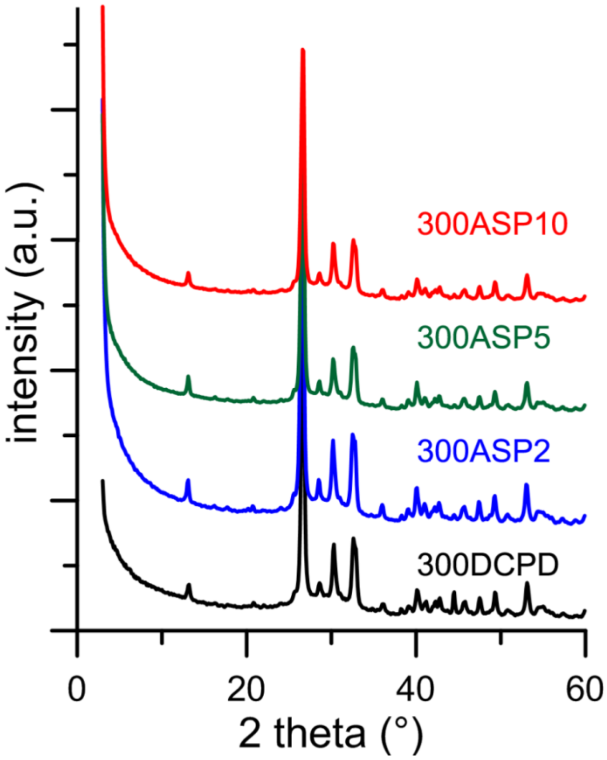

Figure S4: Powder X-ray diffraction patterns of the products obtained from the synthesis of DCPD in the presence of increasing concentrations of ASP after heat treatment at 300 °C.

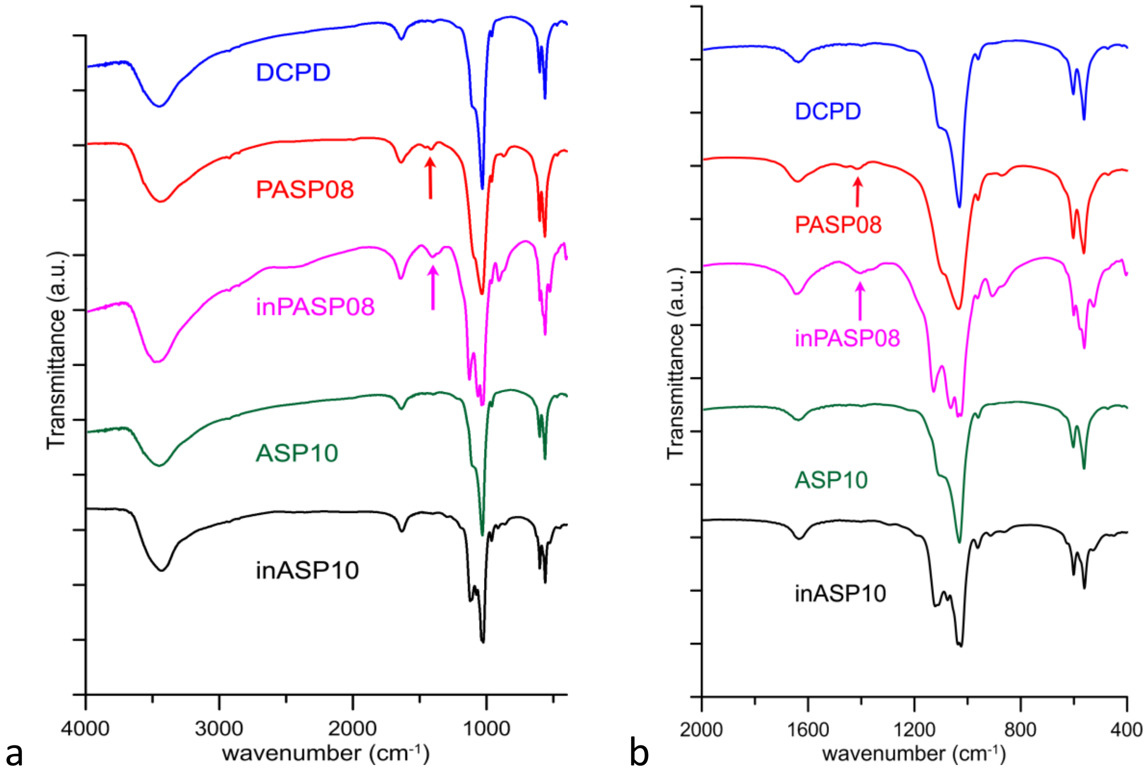

Figure S5: (a) FT-IR spectra of the different samples after 3 days of hydrolysis at 60°C; (b) enlargement of the 400-2000 cm^{-1} part of the spectra.
